# Timing of Colonization of Caries-Producing Bacteria: An Approach Based on Studying Monozygotic Twin Pairs

**DOI:** 10.1155/2011/571573

**Published:** 2011-10-19

**Authors:** Michelle R. Bockmann, Abbe V. Harris, Corinna N. Bennett, Ruba Odeh, Toby E. Hughes, Grant C. Townsend

**Affiliations:** Craniofacial Biology Research Group, School of Dentistry, The University of Adelaide, Adelaide, SA 5005, Australia

## Abstract

Findings are presented from a prospective cohort study of timing of primary tooth emergence and timing of oral colonization of *Streptococcus mutans* (*S. mutans*) in Australian twins. The paper focuses on differences in colonization timing in genetically identical monozygotic (MZ) twins. Timing of tooth emergence was based on parental report. Colonization timing of *S. mutans* were established by plating samples of plaque and saliva on selective media at 3 monthly intervals and assessing colony morphology. In 25% of individuals colonization occurred prior to emergence of the first tooth. A significant proportion of MZ pairs (21%) was discordant for colonization occurring before or after first tooth emergence, suggesting a role of environmental or epigenetic factors in timing of tooth emergence, colonization by *S. mutans*, or both. These findings and further application of the MZ co-twin model should assist in development of strategies to prevent or delay infection with *S. mutans* in children.

## 1. Introduction

Early childhood caries is again on the rise in Australian children despite considerable public health initiatives, including fluoridation of drinking water and use of fluoridated toothpaste. Dental caries continues to affect large numbers of children with nearly 50% of Australian 6-year-olds having a history of decay in their primary teeth and 10% having at least 8 affected teeth [1]. For children under the age of 15 years, dental procedures are the most common reason for undergoing a general anaesthetic in Australia [2].

Dental caries is not only affecting the most vulnerable people in our community, leading to significant human costs of pain, discomfort, and issues of self-esteem, but management of dental caries is also associated with considerable financial cost to individuals and governments. A greater understanding of the behaviour of cariogenic bacteria in the oral environment, together with improved knowledge of the nature of the interplay between a person's genetic makeup and their exposure to environmental factors, should lead to better methods for assessing caries risk and, in turn, establishing more effective prevention strategies.

Caries is recognised as a multifactorial disease as a result of the findings of many studies that have investigated the ecology of dental plaque, including the different types of microflora that may be present, the levels of various oral bacterial species, and also the patterns of microbial transmission observed within families [3–5]. Until recently, relatively little was known about the role of genetic factors in dental caries initiation in humans [6]. Our focus in this paper is to throw new light onto how genetic and environmental factors influence observed variation on the timing of colonization of *Streptococcus mutans* (*S. mutans*) in the oral cavities of a large sample of monozygotic (MZ) twins. *S. mutans* is the most well-documented species of the microbiological genus Mutans streptococci (MS). MS are generally considered to be some of the major pathogens associated with the process of dental caries, and *S. mutans *is frequently isolated from carious lesions [5]. MS exist as part of the oral biofilm's ecosystem, and they are characteristically anaerobic, acidogenic, aciduric, and carbohydrate metabolizers.

The oral microenvironment consists of many different bacteria, and it is the balance of these bacteria that determines both health and disease of the oral tissues. Several models have been proposed to explain the commencement and progression of dental caries. The extended caries ecological hypothesis explains the caries process, comprising a stable stage, an acidogenic stage, and an aciduric stage [5]. There is a shift in bacteria within the oral biofilm from non-MS and actinomyces at the stable stage, to MS and lactobacilli in the aciduric stage, although it is possible to reverse this process [5, 7]. 

Caufield et al. [8] proposed a window of infectivity for the initial colonization of MS coinciding with the emergence of the primary teeth. It was found that the average age of colonization was around 26 months of age, about the time when most of the primary teeth had emerged into the oral cavity. However, some evidence exists supporting MS colonization in predentate individuals [9]. Studies have shown that earlier colonization of MS can lead to an earlier onset of dental caries in children under five years of age [10–12].

Our research group has been conducting dental research involving Australian twins and their families for over 25 years. By using twins we can clarify how genetic and environmental influences affect the timing of dental development and also the timing of colonization of MS within the oral cavity. For example, we have shown already that there is a very strong genetic contribution to the timing of emergence of the primary teeth [13, 14].

In this paper we will focus on the differences rather than the similarities between MZ co-twins, who share a common genetic makeup and often a common environmental background. This should allow us to gain greater insight into unique environmental effects operating on the twins as individuals as well as epigenetic influences [15]. The advantage of MZ twins for these types of studies is that they are matched perfectly for age and sex, and share the same genes.

Given the lack of information on genetic and environmental contributions to variation in MS colonization, the aim of this study is to clarify whether there is a definite pattern of association between the timing of emergence of the first primary tooth and the timing of colonization with *S. mutans* in pairs of MZ twins. We hypothesize that colonization will occur after emergence of the first primary tooth, and also that MZ co-twins should show a similar sequence of first tooth emergence and colonization, reflecting underlying shared genetic influences.

## 2. Methods

### 2.1. Study Cohort

The cohort used in this study is from a larger longitudinal study of twins focusing on primary tooth emergence and oral health [16]. The study sample consists of 151 MZ twin pairs who were recruited into our study between 0 and 1 year of age and are now aged between 2 and 8. The co-twins have all been raised together, are all of European ancestry, and are all in good health. Twins enrolled in this study were recruited through the Australian Twin Registry, Australian Multiple Births Association, newspaper birth announcements, hospitals and prenatal classes. Parents provided informed consent for their twins. 

Zygosity of the MZ twins has been confirmed by DNA analysis of 10 highly polymorphic genetic loci (D3S1358, vWA, FGA, AMEL, D8S1179, D21S11, D18S51, D5S818, D13S317, D7S820) covering 10 chromosomes from buccal swabs. The study sample includes 67 pairs of MZ males and 84 pairs of MZ females. Ethical approval has been obtained by the University of Adelaide Human Research Ethics Committee (H-78-2003).

### 2.2. Recording Methods

Tooth emergence for the twins was determined by parental reports using specially designed recording charts. Parents were given detailed instructions and were advised to note the date when the tooth first broke through the gingival surface and how to palpate for the tooth. The accuracy of parental reports has been confirmed by clinical examination of randomly selected twins aged 3 months to 2 years [13]. Birth weight and gestational age were obtained from the parents via a questionnaire administered before age one, which captures significant developmental time points. This questionnaire consists of questions relating to the conditions surrounding the pregnancy, birth and early months of life of the twins. The parents were asked questions about problems that may have occurred during pregnancy, type of delivery, placenta type, twins' birth weights and lengths, and parental lifestyle habits.

### 2.3. Colonization

Specifically engineered collection kits were mailed to parents quarterly, commencing at 3 months of age, to collect saliva and plaque samples of oral bacteria from the twins. Each kit contained two swabs per person for collection of one morning and one evening sample on a single day. Parents were instructed to wipe over the oral cavity, including the gums and tongue, and also teeth when present, using a sterile cotton swab for approximately 10 seconds. They then placed the swab tip into a sterile ependorf tube containing a semisolid transport medium to ensure the survival of the oral bacteria during transportation. The ependorf tubes were then sealed tightly and posted to the laboratory in Adelaide. Upon arrival, each sample was plated out on selective media (TYS20BA) then incubated for 48 hours at 37°C in an atmosphere of 95% nitrogen and 5% carbon dioxide. After incubation, plates were scored visually under a dissecting microscope for presence or absence of *S. mutans* based on colony morphology. A subsample of colonies identified as positive through visual scoring was confirmed as *S. mutans* by analysis of carbohydrate fermentation patterns. Twins were tested every three months until three contiguous positive scores for both twins were obtained. At least three collection kits were administered to the families covering a period of no less than 9 months. The date at which the first of the three positive scores was identified for each twin was used as their colonization date.

### 2.4. Data Analysis

Descriptive statistics (interval scale variables—means, standard deviations; dichotomous variables—frequencies, relative frequencies) were calculated using one randomly selected twin per pair for all variables. Where variable means are presented in the text, they are accompanied by the sample standard deviation. Intra- and interobserver errors for colonization scoring were very low (Cohen's kappa ~ 0.9).

Sexes were compared using variance ratio (*F*) tests and Student's *t*-tests. The relationships between timing of both first tooth emergence and colonization and between twin pairs for interval scale data were examined using Pearson's correlation coefficient. Intrapair differences were examined using paired *t*-tests (interval scale data).

## 3. Results

Gestational age for the twins ranged from 29 weeks to 40 weeks. Twin pairs considered premature (<37 weeks gestation) comprised 62% of the sample (males 63%, females 61%).

Males (2.5  ±  0.6 kg) were heavier, on average, than their female twin counterparts (2.3 ± 0.6 kg). Optimal birth weight of twins is 2.5 kg or greater, with those individuals less than this classified as either low (1.5–2.5 kg) or very low (1.5 kg and less) birth weight. In our study 46% of males and 62% of females were of low to very low birth weight. Fourteen twin pairs exhibited a birth weight difference of 500 grams or greater.

The first tooth to emerge was generally a lower central incisor, with no evidence of directional asymmetry in emergence times. The first tooth erupted significantly earlier in males (7.8 ± 1.6 months) than females (8.8 ± 2.0 months). Females were also significantly more variable for timing of first tooth emergence.

Table 1 lists the proportion of concordant pairs for emergence of the first tooth, illustrating the trend as a progressively more liberal interpretation of concordance was applied. Allowing for a discrepancy of up to 28 days between co-twins, 86% of the twin pairs were concordant for timing of emergence of the first tooth. Male and female patterns are also presented in the same table.

The mean age of colonization was 12.7 ± 6.1 months, with the earliest time of colonization observed at 2.4 months and the latest to colonize at just over 2.5 years.  Table 2 shows the overall proportion of twin pairs concordant for *S. mutans *presence, and additionally the breakdown of male and female twin pairs. Allowing for a discrepancy of up to 12 months between co-twins, 93% of the 151 twin pairs were concordant for *S. mutans* colonization.

**Figure 1:**
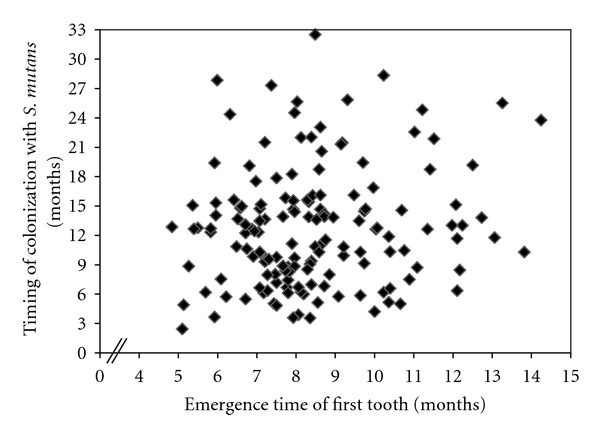
Scatter diagram of timing of colonization against timing of emergence of the first tooth for one randomly selected twin.

Figure 1 examines the relationship between tooth emergence timing and colonization timing. There was no significant association between timing of tooth emergence and timing of colonization. A log transformation of the data did not improve the fit significantly.

Table 3 compares twins within a pair for their colonization status before the emergence of the first tooth. Concordance for colonization prior to first tooth emergence was 15% (23 twin pairs). Concordance for colonization after first tooth emergence was 64% (97 twin pairs). The remaining 21% (31 twin pairs) were discordant.

The covariates, birth weight and timing of first tooth emergence, were not significantly different between pre- and postemergence colonizers, with an average intrapair difference of 0.27 ± 0.24 kg and 18 ± 30 days, respectively. Timing of *S. mutans* colonization was, unsurprisingly, significantly different between pre- and postemergence colonizers, with an average intrapair difference of 7.2 ± 5.2 months.

## 4. Discussion

Studies of twins have contributed significantly to our understanding of the role of genetic factors in the process of dental caries in humans [17–19]. Most previous studies of dental caries based on twins have employed the classical twin model in which comparisons are made between MZ twin pairs who share the same genes and dizygotic (DZ) twin pairs who share 50% of their genes on average. This model enables estimates to be made of the heritability of selected phenotypes, with values ranging from 0% (no genetic contribution to observed variation) to 100% (all the variation can be explained by genetic factors). Different researchers have focussed on different variables relating to the process of dental caries, with evidence of genetic influences being found for bacterial, dietary, and host factors [6, 20, 21]. There is also evidence, based on assessments of the genetic correlation between primary and permanent caries scores, that different genes may be involved in the carious process between dentitions [22]. While estimates of heritability are important in establishing whether there is a significant genetic contribution to phenotypic variation, they are population-based statistics, and caution is needed in extrapolating findings to the individual. For example, even though the estimate of heritability for a given feature may be high, this does not necessarily mean that an environmental intervention cannot have a major effect on the phenotype.

Another twin model that has been applied in a limited way to the study of dental caries in humans is the Twins Reared Apart model. Two studies based on twins in the Minnesota Study of Twins Reared Apart have provided valuable insights into the important role of genetic influences on the carious process [23, 24]. These studies looked at caries experience in adult twin pairs who had been separated around birth and then raised in different environments throughout their lives. Despite their separation, the twin pairs showed remarkably similar patterns of dental caries experience as disclosed by the numbers of decayed, missing and filled teeth. The researchers noted that there were several variables, all of which are likely to have a genetic basis that could explain their findings including: similarities in salivary factors and oral microflora; similarities in timing and sequence of tooth emergence; similarities in dental morphology, arch dimensions, and dental spacing; and dietary preferences. Our previous studies of Australian twins have confirmed that there is a significant genetic contribution to variation in timing of tooth emergence and various morphological features of both the primary and permanent dentitions [13, 14].

The twin model that we have applied in the present study is the MZ co-twin model which has several advantages for studies of complex diseases such as dental caries. For example, MZ co-twins are matched for age and sex and have very similar dentitions from a developmental and morphological perspective, reflecting their similar genetic makeup [16, 25, 26]. We have, however, shown that MZ co-twins are commonly discordant for the expression of certain dental features, such as missing and extra teeth, which reflects differences in environmental and/or epigenetic, influences between the co-twins [15, 27]. The MZ co-twin model therefore provides an opportunity to obtain new insights into the interactions between genetic, epigenetic and environmental influences on phenotypic variation. The MZ co-twin model is extremely powerful because data from only a relatively small number of twin pairs are required to be examined to gain insight. This makes this particular twin model ideal for clinical studies where it is often difficult to recruit the large numbers of subjects who are otherwise required for studies based on the classical twin model.

Our approach to the use of the MZ co-twin model has been to focus initially on the early stages of the carious process, that is, the initial colonization of caries-related microorganisms within the oral cavity. This approach is in contrast to many previously published studies which score the outcomes of the process, that is, decayed missing and filled teeth. It is clear that further studies are needed on genetic contributions to variability observed between individuals at all stages of the process of dental caries. However, we believe that focussing on the early stages may provide results that will have more immediate application in the prevention of the disease. 

By referring to the detailed information on general health, oral hygiene practices, and diet of the twins and their families in our study, we have been able to retrospectively explore potential factors that may have contributed to discordances between MZ co-twins. For example, differences in the timing of initial colonization of decay-producing bacteria such as *S. mutans*, as well as exploring why some twin pairs or co-twins may have become colonized with *S. mutans* prior to the emergence of the first primary tooth and others afterwards. We acknowledge that *S. mutans* is not the only microorganism that is involved in the carious process, and that around 10% of individuals with rampant caries do not have detectable levels of *S. mutans* [28]. We consider, however, that there is sufficient published evidence [20] to focus on genetic and environmental influences relating to this microorganism in the first instance within the context of the ecological plaque hypothesis [29].

Further investigation of significant differences in measurable variables such as biologically meaningful birth weight differences between co-twins creates a unique environmental factor which may be contributing to discordance of other variables. Fourteen twin pairs in our current sample exhibited a birth weight difference of 500g or greater, possibly as a result of twin-twin transfusion syndrome (TTTS) arising from vascular anastomoses *in utero*. Such birth weight discordance may have significant effects on the future health and wellbeing of the lighter twin, as well as implications for the timing and processes of development that scale allometrically with body weight. TTTS complicates traditional twin models as it is a function of the MZ twinning process and hence reduces the MZ correlation relative to the DZ correlation, overestimating the contribution of the unique environment to phenotypic variance.

The mean time of first tooth emergence in this sample was around 8 months of age, similar to our previously reported findings for the larger cohort [13], and significantly later, by approximately two months, than that commonly reported for singletons [30]. Males had an earlier emerged first tooth than females by approximately one month. This is likely to reflect an allometric relationship between tooth emergence timing and body weight as males were also heavier at birth, on average. However, this finding may also reflect fundamental differences in genetic and/or hormonal influences between sexes acting on the twins *in utero* or early postnatally.

As reported in our recent papers [13, 14], emergence of the first tooth has a very high narrow-sense heritability estimate of 87–96%, suggesting that the process of tooth emergence is under strong genetic control within a population. This is not to say that specific environmental (e.g., TTTS) or epigenetic factors cannot give rise to significant discrepancies in tooth emergence timing within individual MZ pairs. When tooth emergence timing in twin pairs in the current study was categorized as concordant/discordant, allowing an intrapair difference of up to 28 days, approximately 90% of the twin pairs were classified as concordant. When taken in light of our previous high estimates of heritability, this suggests that an intrapair difference of greater than one month is appropriate for ascertainment of MZ twins markedly discordant for tooth emergence timing and for further analysis of unique environmental or epigenetic influences.

The mean age of colonization (12.7 ± 6.1 months) calculated for our sample of twins is one of the first large-sample estimates of colonization timing reported in the literature as far as we are aware. At a population level, colonization was both later and more variable than timing of first tooth emergence. Both distributions showed significant overlap, and there was no significant association between timing of first tooth emergence and timing of colonization (see Figure 1). These two factors cast doubt on a model of colonization which requires a hard tooth surface to be present in the mouth prior to colonization, and this is emphasized by the fact that approximately 25% of our the individuals in our sample were colonized prior to tooth emergence. This result supports the work of Wan et al., who showed that colonization can occur in predentate singletons [9]. It is a significant issue that needs to be considered when developing and analyzing models of early childhood caries aetiology.

In a manner analogous to that for timing of emergence of the first tooth, when colonization timing in twin pairs in the current study was categorized as concordant/discordant, allowing an intrapair difference of up to 12 months, approximately 90% of the twin pairs were found to be concordant. When taken in light of our previous moderate- to high estimates of heritability for colonization timing [31], this suggests that an intrapair difference of greater than a year is appropriate for ascertainment of MZ twins markedly discordant for colonization timing for further analysis of unique environmental or epigenetic influences. An exploration of our questionnaire material for feeding practices, tooth brushing habits, and general health may give further insight into factors influencing the timing of *S. mutans *colonization.

The relationship between tooth emergence timing and colonization timing was examined further by comparing twins within pairs for their event sequence (i.e. colonization before or after tooth emergence). A significant proportion (21%) was discordant for this sequence. We have demonstrated that discordance was not due to birth weight discrepancies between twins, nor to marked intrapair differences in tooth emergence timing. It is likely that a range of genetic and nongenetic factors play a significant role in both the timing of emergence of the first tooth and when the oral cavity becomes colonized with* S. mutans*, and further multivariate modelling of this relationship in the larger cohort of twins is ongoing.

A particularly exciting prospect for future studies of dental caries progression will be to carry out genomic and epigenomic scans of the MZ co-twins who are discordant for expression of the disease or for factors known to be linked to the disease. Already, studies have been performed showing that there can be differences in the epigenetic profiles of MZ twin pairs, and that these differences can be associated with discordances in particular phenotypic features between the co-twins [27, 32]. However, so far we are not aware of any studies of this type that are related to dental caries. A recent study has provided the first genome-wide scan for dental caries in a human population [33]. These researchers were able to identify suggestive genetic loci for both low caries susceptibility and high caries susceptibility on chromosomes 5, 13, and 14, as well as on the X chromosome. They proposed that genes related to salivary flow and dietary preferences were possible candidates. They also speculated that there may be a protective locus for caries on the X chromosome that might explain the tendency for a difference in caries experience between the sexes. There has also been a complex segregation analysis carried out recently on Brazilian families that has indicated a dominant, major gene effect influencing resistance to dental caries. We believe that future studies combining the advantages of studying twins and their families with modern methods of genome scanning and segregation analyses offer great potential to identify key genetic risk factors for susceptibility to dental caries.

It has generally been assumed in the past that dental caries is mainly determined by environmental factors, and so most of the strategies for preventing or managing the disease have focussed on modifications to that environment, including oral hygiene or diet alteration. However, dental caries continues to be a major public health issue, even in countries such as Australia [34]. There is a growing interest in the identification of risk factors that might predispose individuals to dental caries and also in identifying factors that might provide individuals with protection. It is highly likely that these factors will reflect the genetic makeup of host-related factors, including the nature of the oral biofilm. If our understanding of the development of the oral biofilm can be improved, it may be possible to adjust its ecology and thereby decrease the likelihood of children developing dental caries. Clinical applications of findings from our project, focussing on preventive practices in young children during primary tooth emergence, promise to lead to reduced dental disease prevalence and significant reductions in health expenditure [3]. Hillman's work with genetically modified mutans has been through the clinical trial stage and our findings on timing of *S. mutans* colonization will provide important evidence for the most appropriate timing of inoculation [35, 36]. Thus, information on colonization timing will be invaluable for developing strategies to prevent or delay infection with MS in young children.

## Figures and Tables

**Table 1:** Twin pair concordance for timing of emergence of the first tooth allowing 0, 7, 14, 21, and 28 days difference between co-twins.

	% concordance		
	All	Males	Females
0 days	14	9	18
7 days	45	49	42
14 days	68	76	61
21 days	79	85	75
28 days	87	90	85

**Table 2:** Twin pair concordance for timing of MS colonization allowing 0, 3, 6, 9, and 12 months difference between co-twins.

	% concordance		
	All	Males	Females
0 months	54	57	51
3 months	66	64	68
6 months	78	78	79
9 months	89	88	89
12 months	93	91	95

**Table 3:** Associations between MZ co-twins (Twin A and B) for colonization prior to emergence of the first tooth.

		Twin A	
		Colonization prior to emergence	
		No	Yes
Twin B	No	97	13
Colonization prior to emergence	Yes	18	23
